# Contribution of Hypothermia and CB_1_ Receptor Activation to Protective Effects of TAK-937, a Cannabinoid Receptor Agonist, in Rat Transient MCAO Model

**DOI:** 10.1371/journal.pone.0040889

**Published:** 2012-07-16

**Authors:** Noriko Suzuki, Motohisa Suzuki, Kazuhiro Hamajo, Koji Murakami, Tetsuya Tsukamoto, Masato Shimojo

**Affiliations:** 1 Pharmaceutical Research Division, Takeda Pharmaceutical Company Limited, Fujisawa, Japan; 2 Division of Pharmaceutical Sciences, Kanazawa University Graduate School of Natural Science and Technology, Kanazawa, Japan; University of South Florida, United States of America

## Abstract

**Background:**

Cannabinoid (CB) receptor agonists are expected to alleviate ischemic brain damage by modulating neurotransmission and neuroinflammatory responses via CB_1_ and CB_2_ receptors, respectively. In a previous study, TAK-937, a novel potent and selective CB_1_ and CB_2_ receptor agonist, was shown to exert significant cerebroprotective effects accompanied by hypothermia after transient middle cerebral artery occlusion (MCAO) in rats. Sustained hypothermia itself induces significant neuroprotective effects. In the present studies, we examined the relative contribution of hypothermia and CB_1_ receptor activation to the cerebroprotective effects of TAK-937.

**Methodology/Principal Findings:**

Using a multichannel brain temperature controlling system we developed, the brain temperature of freely moving rats was telemetrically monitored and maintained between 37 and 38°C during intravenous infusion of TAK-937 (100 µg/kg/h) or vehicle for 24 h after 2 h MCAO. AM251, a selective CB_1_ receptor antagonist, was administered intraperitoneally at 30 mg/kg 30 min before starting intravenous infusion of TAK-937 (100 µg/kg/h) for 24 h. Rats were sacrificed and their brains were isolated 26 h after MCAO in both experiments. When the hypothermic effect of TAK-937 was completely reversed by a brain temperature controlling system, the infarct-reducing effect of TAK-937 was attenuated in part, but remained significant. On the other hand, concomitant AM251 treatment with TAK-937 completely abolished the hypothermic and infarct-reducing effects of TAK-937.

**Conclusions/Significance:**

We conclude that the cerebroprotective effects of TAK-937 were at least in part mediated by induction of hypothermia, and mainly mediated by CB_1_ receptor activation.

## Introduction

TAK-937 has been identified as a structurally novel, selective and highly potent CB_1_/CB_2_ receptor agonist. We reported previously that TAK-937 has dose-dependent cerebroprotective effects in rat transient middle cerebral artery occlusion (MCAO) models [1]. TAK-937 improved not only histological damage in the short term, but also long term (4 weeks) neurological dysfunction and impairment of motor function in a rat transient MCAO model. Furthermore, TAK-937 showed protective effects after embolic MCAO in cynomolgus monkeys [1]. Cerebroprotection by TAK-937 at its optimal doses was accompanied by a decrease in body temperature that is one of the various actions of cannabinoids.

Body temperature is controlled by the hypothalamus, a central locus for thermoregulation. Ischemia of the hypothalamus causes pyrexia. In humans, body temperature in acute stroke is correlated with stroke severity, infarct size, mortality, and functional outcome [2]. Hyperthermia makes brain injury worse. In contrast, hypothermia, even a 1°C decrease, reduces ischemic brain injury [3]. Hypothermia has been shown to be neuroprotective as exemplified by the reduced mortality and improved recovery of cardiac arrest survivors [4], [5] and in neonates after hypoxia/ischemia [6], although the utility of induced hypothermia for the treatment of ischemic stroke patients is not yet established [7]. In our previous study, post-ischemic mild hypothermia that was induced within 4 h after reperfusion showed significant cerebroprotective effects in a rat transient MCAO model [8]. Taken together, it is considered that the hypothermic component of cannabinoid treatment could be a part of their mechanisms for cerebroprotection.

In this study, we determined to what extent hypothermia contributes to cerebroprotection by TAK-937 in a rat transient MCAO model. We also investigated the involvement of CB_1_ receptor in the cerebroprotective and hypothermic effects of TAK-937.

## Results

### Contribution of Hypothermia to Cerebroprotective Effects

**Figure 1:**
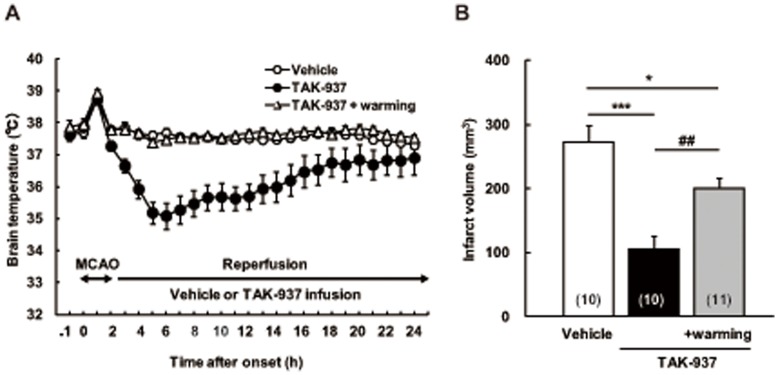
Contribution of hypothermia to cerebroprotective effects of TAK-937 after transient MCAO in rats. Brain temperature (**A**) and infarct volume (**B**). Data are indicated as the means ± SEM. Significant differences from the corresponding vehicle-treated group are indicated by ^***^ *P*<0.001 and ^*^ *P*<0.05 (Dunnett’s test), and from the corresponding TAK-937-treated group is indicated by ^##^ *P*<0.01 (Student’s *t* test). Numbers of rats used are shown in parentheses.

The number of rats assigned to vehicle-treated group, TAK-937-treated group and TAK-937 plus warming group were 12, 14 and 14, respectively. Then, the number of the rats finally adopted to vehicle-treated group, TAK-937-treated group and TAK-937 plus warming group were 10, 10 and 11, respectively, because 9 rats were eliminated by the following reasons: 4 rats died (3 rats in TAK-937-treated group; one rat in TAK-937 plus warming group), one rat in TAK-937 plus warming group did not receive continuous administration appropriately during the experiment, one rat in TAK-937-treated group showed subarachnoid hemorrhage (SAH), one rat in vehicle-treated group did not give continuous data on brain temperature because the telemetry probe was not fixed firmly, and 2 rats had no infarction in the striatum (one rat in vehicle-treated group; one rat in TAK-937 plus warming group). The time course of brain temperature changes in TAK-937, TAK-937 plus warming, and vehicle-treated groups are shown in Fig. 1 (A). TAK-937 (100 µg/kg/h) lowered brain temperature to about 35°C, but the vehicle-treated group rats showed no significant change. The hypothermic effect of TAK-937 was completely reversed to the level of brain temperature in the vehicle-treated group by warming the rats with heating lamps. The infarct volumes measured 1 day after MCAO in TAK-937, TAK-937 plus warming, and vehicle-treated groups were 105.7±20.5, 200.8±16.3, and 272.8±25.6 mm^3^, respectively (Fig. 1 (B)) indicating that TAK-937 significantly reduced the cerebral infarct volume and that the infarct-reducing effect of TAK-937 was attenuated in part by warming, although significant reduction in cerebral infarct volume was still observed.

### Contribution of CB_1_ Receptor Activation to Cerebroprotective Effects

**Figure 2:**
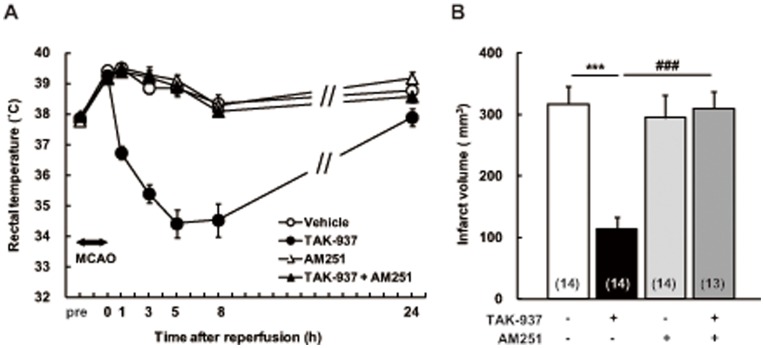
Reversal of cerebroprotection of TAK-937 by AM251, a CB_1_ antagonist, after transient MCAO in rats. Rectal temperature (**A**) and infarct volume (**B**). Data are indicated as the means ± SEM. Significant differences from the corresponding vehicle-treated group are indicated by^ ***^ *P*<0.001 (Dunnett’s test), and from the corresponding TAK-937-treated group is indicated by ^###^ *P*<0.001 (Student’s *t* test). Numbers of rats used are shown in parentheses.

The number of rats assigned to each treatment group was 16. Then, the number of the rats finally adopted to vehicle-treated group, TAK-937-treated group, AM251-treated group and TAK-937 plus AM251-treated group were 14, 14, 14 and 13, respectively, because 9 rats were eliminated by the following reasons: 5 rats died (one rat in vehicle-treated group; one rat in TAK-937-treated group; one rat in AM251-treated group; 2 rats in TAK-937 plus AM251-treated group), one rat in TAK-937 plus AM251-treated group was not injected AM251 appropriately, 2 rats showed SAH (one rat in TAK-937-treated group; one rat in AM251-treated group), and one rat in vehicle-treated group had no infarction in the striatum. The time course of rectal temperature is shown in Fig. 2 (A). TAK-937 significantly decreased the rectal temperature, whereas the vehicle-treated group rats showed no significant change. The hypothermic effect of TAK-937 was completely reversed by AM251, a selective CB_1_ receptor antagonist. The infarct volumes measured 1 day after MCAO in the TAK-937, AM251, TAK-937 plus AM251, and vehicle-treated groups were 114.3±18.4, 294.9±36.8, 309.8±27.3, and 317.0±28.9 mm^3^, respectively (Fig. 2 (B)) indicating that TAK-937 significantly reduced the infarct volume. However, AM251 alone did not change the infarct volume, and the infarct-reducing effect of TAK-937 was completely inhibited by AM251.

## Discussion

In a previous study, TAK-937 was shown to reduce infarct volumes after transient MCAO at doses where hypothermia was induced [1]. However, hypothermia itself exerted strong neuroprotection. In the present study, we sought to examine the contribution of hypothermia and CB_1_ receptor activation to the protective effects of TAK-937 on cerebral infarction after transient MCAO in rats, to investigate the mechanism of the cerebroprotective effects of TAK-937.

To maintain a constant body temperature in animals, conventional apparatus such as a conductive water mattress, convective air warmer, and heating lamp are used in anesthetic conditions where the body temperature drops generally. However, it is difficult and impractical for that type of apparatus to be used with conscious, freely moving animals. Therefore, we have developed a multichannel brain temperature controlling system to maintain a constant brain temperature of conscious rats strictly in a range between 37 and 38°C by modification of our original brain temperature controlling system used to induce mild hypothermia [8] referring to the method of DeBow and Colbourne [9]. As shown in Fig. 1 (A), brain temperature of control rats was kept constant throughout the drug administration period. Using this system, we revealed that the cerebroprotective effect of TAK-937 was partially attenuated by reversing hypothermia to normothermia. However, significant protection by TAK-937 was still observed in this condition. This finding suggested that the cerebroprotective effects of TAK-937 are mediated in part by induction of hypothermia. The present finding that the cerebroprotective effect of TAK-937 was seen without hypothermia is consistent with our earlier finding that TAK-937, at 10 µg/kg/h where hypothermia was not seen, significantly reduced infarct volume after transient MCAO. Thus, it was suggested that mechanisms other than hypothermia would be involved in cerebroprotective effects of TAK-937. In this experiment, we simultaneously measured brain temperature telemetrically and rectal temperature manually at some time points (Table 1), because it was said that rectal temperature might not accurately reflect brain temperature especially during global ischemia. As a result, we confirmed that there were little difference (about 0.5°C) between brain temperature and rectal temperature in the experiment, and both temperature changed to parallel. This observation is supported by the report showing positive correlation (r = 0.91) between brain temperature and rectal temperature in the same model as ours [10]. Based on this finding, we measured just rectal temperature in the experiment of contribution of CB_1_ receptor activation.

**Table 1:** Effect of TAK-937 on rectal and brain temperature in rats with transient MCAO.

Paramater	Groups	No. of		Time after drug administration (h)			
		animals	Before	0	3	5	24
Brain	Vehicle	10	36.7±0.2	38.7±0.2	37.7±0.2	37.6±0.1	37.4±0.1
Temperature	TAK-937	10	36.9±0.2	38.7±0.2	35.6±0.4	35.2±0.4	36.9±0.5
(°C)	TAK-937+ warming	11	36.9±0.2	38.9±0.1	37.5±0.1	37.5±0.1	37.6±0.1
Rectal	Vehicle	10	37.3±0.1	39.4±0.1	38.2±0.1	38.2±0.1	37.9±0.1
temperature	TAK-937	10	37.3±0.1	39.4±0.1	35.5±0.4	35.0±0.4	36.9±0.4
(°C)	TAK-937+ warming	11	37.4±0.1	39.3±0.1	37.7±0.1	37.8±0.1	37.8±0.1

To pharmacologically reverse the hypothermic effect of TAK-937, a CB_1_ receptor antagonist was applied because it has been reported that the hypothermic effect of cannabinoids is mediated by CB_1_ receptors and not CB_2_ receptors [11]. As shown in Fig. 2 (A), the hypothermic effect of TAK-937 was completely reversed by concomitant administration of AM251, a selective CB_1_ receptor antagonist. Moreover, the cerebroprotective effect of TAK-937 was also completely reversed, in contrast to the physical reversal of hypothermia. This result suggested that the cerebroprotective effect of TAK-937 is almost mediated by CB_1_ receptor agonist activity, and mechanisms of cerebroprotection by TAK-937 other than hypothermia may be also mediated by the CB_1_ receptor.

So far, several studies have investigated the contribution of hypothermia to neuroprotection by several known CB receptor agonists. HU-210 demonstrated a dose-dependent reduction in infarct volume after focal cerebral ischemia, and cerebroprotection was associated with indirect protective effects of hypothermia [12]. WIN 55,212-2-induced hypothermia (to approximately 34°C) demonstrated significant effects in reduction of infarct volume after focal and global cerebral ischemia, and this cerebroprotection was still observed after warming the animals to reverse their body temperature to the same level as that of control animals [13]. Thus, our study further confirmed the involvement of hypothermia in the cerebroprotective effect of CB receptor agonists and clearly indicated that other mechanisms mainly mediated via CB_1_ receptors exist.

The mechanisms underlying the protective effects of CB receptor agonists unrelated to hypothermia have been shown in several in vitro studies. HU-210 and WIN 55,212-2 significantly reduced excitatory postsynaptic currents in a dose-dependent manner and inhibited the synaptic release of glutamate in rat dorsolateral striatal brain slices [14]. CP 55,940 and WIN 55,212-2 remarkably reduced hippocampal cell death in cultured neurons subjected to high levels of NMDA, and WIN 55,212-2 also inhibited the NMDA-induced increase in intracellular calcium concentration by initiating CB_1_-mediated inhibition of adenylate cyclase [15]. Furthermore, CP 55,940 inhibited the release of nitric oxide (NO) from rat microglial cells via CB_1_ receptor, which may play an important role in the central nervous system to elicit immune-mediated neurodegenerative inflammatory processes and cause brain injury [16]. These mechanisms may act additively or synergistically with hypothermia. It seems prudent to investigate whether TAK-937 also exerts those actions to fully understand the mechanisms of its cerebroprotective effects.

In addition, we cannot yet neglect the potential contribution of CB_2_ receptor agonist activity to the TAK-937’s cerebroprotective effects, because contribution of CB_2_ receptor activation could be masked by strong CB_1_ receptor activation. In order to examine the involvement of CB_2_ receptor activation, we tried to determine the appropriate doses and dosing schedule for CB_2_ receptor antagonist to block CB_2_ receptor activation certainly utilizing some biomarkers as we utilized body temperature in the case of CB_1_ receptor activation. Anti-inflammatory effects of CB_2_ receptor activation by examining the changes in pro-inflammatory cytokines such as interleukin-1β (IL-1β) and tumor necrosis factor α (TNF-α) are considered as PD markers [17]. We explored the potential for utility of those pro-inflammatory cytokines as PD markers using AM630, a CB_2_ receptor antagonist [18], [19], but we could not identify the appropriate experimental conditions to examine the involvement of CB_2_ receptor activation in our model. In other words, substantial doses of AM630 did not block suppressive effects of TAK-937 on cytokine elevation (data not shown). Several literatures reported that CB_2_ receptor involved in microglial/macropharge activation, and influenced on alternative mediators/markers such as IL-10 and transforming growth factor β (TGF-β) [20]. Future studies are required to determine the appropriate dosing using PD markers such as IL-10 or TGF-β to clarify the involvement of CB_2_ receptor activation.

Therapeutic hypothermia has been well established in experimental animals [8], [21] and is applied clinically to patients with cardiac arrest [4], [5]. On the other hand therapeutic hypothermia is not widely applied in acute ischemic stroke [7]. Several clinical trials to assess the efficacy of hypothermia on acute ischemic stroke are currently underway [22], [23]. One of the reasons why therapeutic hypothermia is not so widespread is considered to be its inconvenience. Hypothermia with mechanical cooling provokes strong physiological counter responses such as vasoconstriction, shivering, hypertension, and tachycardia. Those responses should be cautiously monitored in well-organized facilities that provide specialist instruments and expert clinical support, such as in intensive care units, resulting in expensive treatment. In contrast, TAK-937 did not change blood gas parameters (PaO_2_, PaCO_2_, or pH) in a rat MCAO model and neither mean arterial pressure nor heart rate at doses effective in a cynomolgus monkey embolic MCAO model, although TAK-937 induced hypothermia in both models [1]. Thus, drug induced hypothermia using TAK-937 appears to be superior to mechanical cooling.

In conclusion, we found that the cerebroprotective effects of TAK-937, a novel potent and selective CB_1_ and CB_2_ receptor agonist, were mediated partially by induction of hypothermia and mainly by CB_1_ receptors. TAK-937 promises to be a beneficial therapeutic agent for treatment of acute ischemic stroke patients.

## Materials and Methods

### Experimental Animals

#### Ethics statement

The animal care and all the experimental procedures were conducted in accordance with guidelines approved by the Animal Use Ethical Committee of Takeda Pharmaceutical Company Limited, Japan (Approval ID: TEACUC-2446 and TEACUC-2711).

Eight-week-old male Sprague Dawley rats (CLEA Japan Inc., Tokyo) weighing 300 to 330 g were purchased at 7 weeks of age, and acclimated for about 1 week prior to surgery. The rats were housed in groups of 5 per cage in an ambient temperature of 24±1°C and humidity of 55±5% in a light-controlled room (12 h light/dark cycle with lights on at 07:00 h) and were given water *ad libitum*, and fed a commercial diet.

### Drugs

TAK-937, *N*-[(3*R*)-7-[(1*R*)-1-Hydroxyethyl]-3-(4-isopropylphenyl)-4,6-dimethyl-2,3-dihydro-1-benzofuran-5-yl]-3,3-dimethylbutanamide (Lot No. B21567-076-04) was synthesized at Chemical Development Laboratories, Takeda Pharmaceutical Company Limited. TAK-937 was solved in 5% hydroxypropyl-β-cyclodextrin (HPBCD). AM251, *N*-(Piperidine-1-yl)-5-(4-iodophenyl)-1-(2,4-dichlorophenyl)-4-methyl-1H-pyrazole-3-carboxamide (Lot No. B18158-068-14), a selective CB_1_ receptor antagonist, was synthesized at the Medical Chemistry Research Laboratories of Takeda Pharmaceutical Company Limited. AM251 was suspended in 0.5% methylcellulose.

Middle cerebral artery occlusion (MCAO).

Transient intraluminal MCAO was induced according to the methods of Koizumi et al. [24] and Zea Longa et al. [25] with modification [26]. Briefly, under halothane anesthesia, a midline incision was made in the neck and the carotid artery bifurcation was exposed. The branches of the external carotid artery were dissected. Next, a silicon-coated nylon suture was inserted via the proximal external carotid artery into the internal carotid artery until its tip reached the origin of the MCA, which was detected by mild increase in resistance. After inserting the suture, rats were allowed to awaken from anesthesia and were returned to their cages. Then, 2 h after MCAO, its success was judged by the appearance of hemi-paresis and rats were reanesthetized with halothane, and reperfusion was allowed by withdrawal of the suture. During the MCAO, the neurological symptoms of each rat were evaluated, and rats showing no hemiplegia were excluded from the experiment. During surgery and MCAO reperfusion, body temperature was monitored, but not controlled.

### Contribution of Hypothermia to Cerebroprotective Effects

In an experiment to assess the contribution of hypothermia to the neuroprotective effects of TAK-937, body temperature was controlled using a multichannel brain temperature controlling system (HEM software, Notocord Systems, Croissy, France) according to the methods of Ohta et al. [8], and DeBow and Colbourne [9] with modification. One day before MCAO, the left femoral vein of rats was catheterized with a polyethylene tube for continuous administration of TAK-937 or vehicle. Subsequently, rats were implanted with a brain telemetry probe (Model XM-FH-BP; Mini-Mitter Co., Bend, OR) as follows. Under halothane anesthesia, the rats were moved to a stereotaxic frame (David Kopf Instruments, Tujunga, CA) and a midline scalp incision was made. In the skull, a small burr hole was drilled 2 mm left of Bregma. The brain telemetry probe covered with a protector was lowered 4 mm below the skull surface and placed into the hole using dental cement. By this method the temperature in the contralateral cortex or corpus callosum was measured. Anesthesia was discontinued and rats were housed individually in cages resting upon the telemetry receivers (RPC-1, DataSciences Int., St. Paul, MN) that continuously sampled brain temperature. On the next day, transient MCAO was produced as described above. Rats were returned to their cages upon the telemetry receivers again during MCAO. The intravenous administration of TAK-937 or vehicle was started just after the reperfusion. TAK-937 was administered at a dose of 125 µg/kg/10 min, followed by 100 µg/kg/h for 24 h. A solution of 5% HPBCD was administered as vehicle control at the same administration rate as TAK-937. Using a multichannel brain temperature controlling system, the brain temperature of freely moving rats was telemetrically monitored and maintained between 37 and 38°C for 24 h after reperfusion of the vehicle-treated and TAK-937-treated and warmed groups as follows. For the vehicle-treated group, two cooling fans equipped on the cage lid were turned on through an analogue feedback circuit and when the brain temperature became higher than 38°C; the fans were stopped when the brain temperature reached below 37°C. For the TAK-937-treated and warmed group, an infrared heat lamp was turned on through an analogue feedback circuit when the brain temperature became lower than 37°C; and the lamp was stopped when the brain temperature reached over 38°C. For the TAK-937-treated group, rats were kept at room temperature without controlling brain temperature. The brain temperature was recorded every 2 min for 2 sec on a receiver connected to a computer with Dataquest A.R.T. System (DataSciences Int., St. Paul, MN). The rectal temperature was measured before MCAO, just before drug administration, and 3, 5 and 24 h after drug administration with an electronic digital thermometer (BAT-12, Physitemp Instruments, Clifton, NJ). Infarct volumes were determined 24 h after administration, as detailed below.

### Contribution of CB_1_ Receptor Activation to Cerebroprotective Effects

One day before MCAO, the left femoral vein was catheterized with a polyethylene tube for continuous administration of TAK-937 or vehicle. TAK-937 was dissolved in 5% HPBCD as vehicle. On the next day, transient MCAO was induced as described above. AM251 at 30 mg/kg or vehicle was intraperitoneally injected at 2 mL/kg 30 min before the reperfusion. The intravenous administration of TAK-937 or vehicle was started just after the reperfusion. TAK-937 was administered at a dose of 125 µg/kg/10 min, followed by 100 µg/kg/h for 24 h. Solution of 5% HPBCD was administered as vehicle control at the same administration rate as TAK-937. The rectal temperature was measured before MCAO, just before drug administration, and 1, 3, 5, 8, and 24 h after drug administration with an electronic digital thermometer (BAT-12, Physitemp Instruments, Clifton, NJ). Infarct volumes were determined 24 h after administration, as detailed below.

### Measurement of Infarct Volume

Twenty-four hours after administration, rats subjected to MCAO were killed and then their brains were quickly removed and chilled in ice-cold saline. Six 2 mm thick, coronal slices were cut with a brain slicer (Neuroscience Idea, Osaka, Japan). The coronal slices were incubated in a saline solution containing 1% 2,3,5-triphenyltetrazolium chloride (TTC; Wako Pure Chemicals, Osaka, Japan) at 37°C for 15 min. After TTC staining, the images of the coronal slices were taken with a digital camera. The images were analyzed to quantify the infarct area with Adobe Photoshop software and infarct volume was determined by the integration of the infarct area of each slice and the distances between them.

### Statistical Analysis

All data were expressed as means ± standard error of mean (SEM). Statistical analysis between two groups was assessed using a two-tailed, unpaired Student’s *t* test. Differences among three or four groups were analyzed using a one-way analysis of variance (ANOVA) followed by Dunnett’s test. *P*<0.05 was considered to be significant. All statistical analyses were performed using SAS Preclinical Package Version 5.0 (SAS Institute, Japan).
